# The role of dietary tracking on changes in dietary behaviour in a community-based diabetes prevention and management intervention

**DOI:** 10.1017/S1368980025000436

**Published:** 2025-03-27

**Authors:** Ranjita Misra, Delores James

**Affiliations:** 1 School of Public Health, Department of Social & Behavioral Sciences, 3812B, Robert C Byrd Health Science Center, West Virginia University, Morgantown, WV 26506-9190, USA; 2 Department of Health Education and Behavior, College of Health and Human Performance, University of Florida, 1864 Stadium Road, Suite 5, PO Box 118210, Gainesville, FL 32611-8210, USA

**Keywords:** Nutrition education, Dietary tracking, Dietary self-efficacy, Rural, Diabetes mellitus, Prediabetes

## Abstract

**Objective:**

The study examined the impact of the Diabetes Prevention and Management programme on dietary tracking, changes in dietary behaviour, glycosylated Hb (HbA1c) and weight loss over 6 months among rural adults with type 2 diabetes and prediabetes. The programme was a health coach (HC)-led, community-based lifestyle intervention.

**Design:**

The study used an explanatory sequential quantitative and qualitative design to gain insight on participant’s dietary behaviour and macronutrient consumption as well as experience with food tracking. Five of the twenty-two educational sessions focussed on dietary education. Participants were taught strategies for healthy eating and dietary modification. Trained HC delivered the sessions and provided weekly feedback to food journals.

**Participants:**

Obese adults with type 2 diabetes or prediabetes (*n* 94) participated in the programme and 56 (66 %) completed dietary tracking (optional) for 6 months. Twenty-two participated in three focus groups.

**Results:**

Fifty-nine percent consistently completed food journals. At 6 months, average diet self-efficacy and dietary intake improved, and average weight loss was 4·58 (sd 9·14) lbs. Factors associated with weight loss included attendance, consistent dietary tracking, higher HbA1c, diabetes status and energy intake (adjusted *R* ^2^ = 43·5 %; *F* = 0·003). Focus group participants reported that the programme improved eating habits. The consistency of dietary tracking was cumbersome yet beneficial for making better choices and was key to being honest.

**Conclusions:**

Participants who consistently tracked their diet improved dietary self-efficacy and intake over 6 months. This model has the potential to be reproduced in other rural regions of the United States.

West Virginia (WV) is a predominantly rural state with high rates of type 2 diabetes (T2D) (15·9 %) and prediabetes (15·9 % and 34·8 %, respectively)^(1)^. Additionally, high rates of poverty, obesity (41·2 %)^(2)^, poor dietary habits and physical inactivity in the state increase health risks and chronic disease prevalence and complications among adults^(3)^. Lifestyle interventions^(4)^, notably, exercise and dietary education, can significantly delay the onset of T2D and its complications, improve insulin sensitivity and reduce fasting glucose levels^(4,5)^. These T2D programmes also enhance dietary knowledge and practice^(6)^ to improve dietary habits such as consumption of fruits and vegetables, lower salt and fat intake and increase confidence and skills for dietary changes^(7)^. In addition, the American Diabetes Association’s (ADA’s) Nutrition Review Committee recommends a healthy eating plan to improve glycaemic control with relatively fewer side effects and complement their medical management^(8)^. Dietary tracking is a useful tool for encouraging participants to adopt healthier eating behaviours such as estimating portion sizes, consuming food and achieving dietary goals. Additionally, studies note improvements in insulin sensitivity, weight management and HbA1c, leading to a lower risk of microvascular complications and CVD among individuals with T2D or prediabetes^(6,9)^.

Lifestyle modifications have been shown to reduce the need for medications for adults with diabetes. However, approximately half have poor self-care^(10)^ and do not receive provider counselling for behavioural modification^(11)^. Additionally, suboptimal social determinants of health factors (e.g. poor or lack of health care, food insecurity and lack of transportation)^(12)^ in WV impede access to individualised medical nutrition therapy and diabetes self-management education and support (DSMES). Hence, 91 % of WV counties are designated as medically underserved^(13)^. Notably, diabetes is often considered a family or community disease in the region known for disease disparities and poor health outcomes^(14,15)^.

Traditionally, individuals with diabetes and prediabetes have different educational protocols. However, the current diabetes prevention and management programme (DPM) combined two evidence-based programmes – the National Diabetes Prevention Program and the Association of Diabetes Care and Education Specialists self-care behaviours. Additionally, the programme design and implementation were informed by social cognitive theory^(16)^. The DPM^(17,18)^ was a 12-month multicomponent behavioural intervention focused on knowledge, skills, behaviour modification strategies and weight loss in rural adults with T2D or prediabetes in WV^(5,12,19)^. It is important to note that dietary recommendations are the same for both T2D or prediabetes.

As part of a larger study, this article addresses a critical knowledge gap in the dietary intake of rural adults with T2D or prediabetes. Despite widespread interest in evidence-based diabetes nutrition assessments, there is a gap in research on food journaling to track dietary behaviour among rural adults with chronic conditions. In addition, the journaling duration for healthy eating outcomes has not been examined due to the educational support needed for an individual that takes into account personal food preferences, accessibility and sociocultural factors^(20,21)^. Hence, quantitative and qualitative assessments can provide a better understanding of eating behaviour, motivation and engagement. Therefore, the objectives of this study were to (1) evaluate the impact of the DPM programme on food tracking and dietary changes in the first 6 months among obese adults with T2D or prediabetes, (2) describe the baseline dietary behaviour and mean nutrients composition, (3) assess feedback about the programme and experience with food tracking and (4) examine the association of food tracking, dietary behaviour, HbA1c and weight loss over a 6-month programme period.

## Methodology

### Study design and participants

The study used an explanatory sequential quantitative and qualitative design to gain insight into the pre- and 6-month quantitative assessment of participants’ dietary behaviour and macronutrient consumption as well as qualitative focus groups on the feedback and experience with tracking food intake. Data were collected in 2016–2018 from two cohorts of participants (*n* 94) who joined the programme sequentially in 2015–2017. Recruitment flyers were posted in churches, diabetes clinics/hospitals, YMCA and educational institutions. In addition, the investigative team offered information meetings at several local churches, service organisations (e.g. Rotary club) and hospital/clinic diabetes meetings. The study was also advertised in the local newspapers. Eligibility included age 18 years and older, overweight or obese status (BMI ≥ 25 kg/m^2^) and a diagnosis of prediabetes or T2D. The study included 94 adults who screened for eligibility and enrolled in the programme. Cohort 1 participated from August 2015 to July 2016 and completed the baseline, mid (6 months), and end-of-programme (12 months) assessments. Cohort 2 participated from August 2016 to July 2017 and completed all assessments. The study was conducted according to ethical guidelines, and the Institutional Review Board approved all procedures for this research study at a large public university. All participants provided written informed consent before their baseline assessment and programme participation. In addition, all participants were invited to participate in focus groups, and twenty-two individuals accepted. Three focus groups were conducted by two trained qualitative researchers who consulted on the project. Participants provided qualitative feedback about the overall programme and experience with dietary tracking.

### Lifestyle intervention

The DPM programme was a community-based, 12-month, twenty-two-session culturally adapted lifestyle intervention. The programme was an adaptation of the evidence-based curriculum of the National Diabetes Prevention Program^(22)^ and the Association of Diabetes Care and Education Specialists (ADCES7)^(23)^. The intervention was culturally tailored using an advisory board and was implemented in churches in two large counties in WV. The intervention was modelled after the Diabetes Prevention Program and included 60-minute group educational sessions for 12 consecutive weeks, biweekly sessions for 2 months, and monthly sessions for the last 6 months. Trained health coaches (HC) delivered the educational sessions and provided weekly feedback to food journals and health coaching to participants. Programme overview and HC training have been described elsewhere^(24)^. Briefly, HC were students enrolled in professional programmes such as Public Health, Nursing, Pharmacy, Medicine, Physical Activity and Sports Sciences, Exercise Physiology and Human Nutrition. All HC completed 16 hours of training provided by a multidisciplinary team. The training familiarised them with the curriculum, delivering the educational sessions, health coaching and data collection. The programme was implemented in the evening hours during the week (17.30–18.30) or during the weekend (Sunday 13.30–14.30) based on participant preferences. Each participant was assigned an HC who assisted with goal setting and weekly follow-up to identify behaviour modification goals and review strategies (average of 10–15 min) via phone calls, emails and texts (based on participant preference). These discussions provided the opportunity to answer questions, provide continuous feedback on the initiation and maintenance of health behaviours and reinforce health education messages.

Details of the DPM group educational sessions are presented in Table 1. Five of the twenty-two sessions were focused on dietary education. Other DPM programme sessions focused on physical activity, stress management and coping, blood sugar monitoring and problem-solving and staying motivated for a healthy lifestyle. Dietary education focussed on strategies for healthy eating and dietary modification. The session contents included macronutrients, portion size, food label reading, healthy eating and dietary tracking principles, goal setting, meal planning, portion control, mindful eating, tips for healthy eating and energy and macronutrients counting. Two cooking demonstrations were part of the educational sessions (Table 1) and were interactive with taste-testing and skill-building exercises that emphasised key concepts from the educational sessions. It also provided helpful tips, substitutions for ingredients, recipes, food safety and nutritional information. Each session encouraged participants to set realistic, short-term goals and healthy behaviours toward dietary modifications for the week. The programme also encouraged participants to keep daily food journals. The HC provided written (tailored) feedback recognising positive changes, providing general encouragement and discussing additional easy and pragmatic ways to make healthier choices. Participants received self-help educational materials, food journals, a *CalorieKing Calorie, Fat & Carbohydrate Counter* book to measure energies and macronutrients^(25)^, a healthy eating guide, a physical activity guide and a pedometer. Weekly sessions included weigh-ins, group sharing, and problem-solving. At the 6-month assessment, participants who submitted at least 50 % of weekly food journals received a detailed nutrient analysis and counselling to improve their dietary habits.

**Table 1.** DPM programme group education sessions facilitated by health coaches

Month	Schedule	Core sessions^*^	Modules
1	Weekly	1 Welcome	Welcome to the programme
	Weekly	2 Dietary education	Be a fat and energy detective
	Weekly	3 Dietary education	Healthy eating
			Cooking demonstration
	Weekly	4 Physical activity education	Move those muscles
2	Weekly	5 Dietary and physical activity education	Tip the energy balance
	Weekly	6 Dietary education	Take charge of what’s around you
			Cooking demonstration
	Weekly	7 Dietary and physical activity education	Problem solving
	Weekly	8 Dietary education	Four keys to healthy eating out
3	Weekly	9 Dietary and physical activity education	Slippery slope of lifestyle change
	Weekly	10 Physical activity education	Jump start your activity plan
	Weekly	11 Dietary and physical education	Make social cues work for you
	Weekly	12 Dietary and physical activity education	Ways to stay motivated
4	Biweekly	13 Diabetes prevention and management	Monitoring and reducing risks
	Biweekly	14 Dietary education	More volume, fewer energies
5	Biweekly or Monthly	15 Diabetes prevention and management	Prepare for long term self-management
6	Biweekly or Monthly	16 Physical activity education	Strengthen your exercise programme
7	Monthly	17 Dietary education	Mindful eating
8	Monthly	18 Stress management	Stress and time management
9	Monthly	19 Physical activity education	Standing up for your health
10	Monthly	20 Diabetes prevention and management	Heart health
11	Monthly	21 Physical activity education	Stretching: the truth about flexibility
12	Monthly	22 General education	Looking back and looking forward

* Diabetes Prevention and Management (DPM) programme sessions.

### Sample size

The sample size was based on the primary outcome of HbA1c. Ninety participants would provide a clinically meaningful change (0·5) in HbA1c % at 12 months with 80 % power. We estimated a 20 % dropout rate and hence needed seventy-two participants. The initial goal for this paper was to compare changes at 6 and 12 months from baseline. Dietary tracking was recommended but not required for the programme. In addition, food journals had low completion rates during the monthly educational sessions (sessions 17–22). Hence, we used the 6-month assessment for the most completed food tracking data in our analysis. Our estimated sample size for HbA1c change at 6 months showed no change in the number of participants. Although eighty-five participants completed the programme, 56 participants (66 %) completed dietary tracking for 6 months.

### Data collection and measures

Baseline and 6-month data were collected at the intervention sites from 07.00 to 10.00. HC completed anthropometric measurements that included height, weight and waist circumference. Surveys were completed by participants, but HC assisted with surveys as needed. Phlebotomists collected fasting blood for labs. A $25 grocery gift card was provided for completing each assessment.

#### Dietary tracking

Tracking was measured using weekly food journals with columns describing food, type, amount, fat, protein, carbohydrate and energy content. The first 6 months of food tracking were used for nutritional assessments and dietary behaviour for several reasons. First, the programme moved to monthly educational sessions for session 17–22. This resulted in participants receiving monthly feedback on their food journals from HC (instead of weekly). Second, there was a lower return of food journals, probably due to tracking fatigue, which is tedious and time-consuming^(4)^. Third, there was perceived probable confidence for dietary tracking after completing it for 6 months. The HC provided feedback on easy and pragmatic ways to make healthier choices. Participants incorporated continuous feedback into their goals and created new action plans for a healthier diet such as replacing soda and iced tea with non-energy beverages such as water, diet soda and low-fat dressing. Preliminary screening of food journals included checking for completeness. If journal items were not detailed enough, HC requested clarity for quantity and ingredients and resolved any ambiguities at their weekly follow-up discussion. Sixty-six percent (*n* 56) of participants consistently turned in weekly food journals for review and feedback. The participants were categorised as consistent trackers if they completed at least 75 % of their weekly food journal in the first 6 months of the study period. There were no significant demographic differences between participants who completed the food journals *v*. non-trackers (*P* > 0·05). The nutritional analysis was limited to macronutrients (carbohydrates, fibre, total fat, saturated fat, protein), total energies, Na and vitamin D.

#### Diet Self-efficacy

Diet Self-efficacy was measured by a validated twenty-item Eating Habits Confidence survey designed to assess participants’ confidence in their ability to change their eating habits^(26)^. Participants were asked to report how sure they were that they could perform various behaviours on a 5-point Likert-type scale from 1 (I know I cannot) to 5 (I know I can), with the additional option to mark, ‘Does not apply.’ Examples of items were ‘Avoid adding salt at the table’ and ‘Eat poultry and fish instead of red meat at dinner.’ Items were summed for a final score ranging from 0 to 100, with higher scores indicating higher dietary self-efficacy. Cronbach alpha (0·88) deemed it a reliable measure.

#### Dietary intake

Several self-reported diet questions from the FFQ^(27)^ were used to estimate participants’ dietary behaviour at baseline and 6 months. These included how many servings of vegetables and combined fruit and vegetables (per month, per week, per day) they consumed in the previous 6 months. The term ‘fruit’ included fresh, frozen, juices and ‘vegetables’ referred to vegetables, leguminous plants and root vegetables (fresh, frozen, canned, etc.) but not potatoes. To assess high fat intake, the respondents were asked to respond to an adapted question on the frequency (in percentage) of deep-fried food they consume, with five possible options < 5 %, 5–9 %, 10–14 %, 15–25 % and > 25 %. The response to this question was simplified since many questionnaires provide detailed scoring based on consumption of fried food in a typical week, preparation method and portion size based on ≥ 6 times/day to days/weeks/months. Adaption to the question was due to the high rate (15 %) of food insecurity that limits access to healthy foods in WV^(28)^ as well as a lack of variation in the daily consumption of foods, including fried foods. Thus, we categorised the question into percentages assuming that fried foods were deep-fried (*v*. pan-fried) and most consisted of fried meat and potatoes.

#### Attendance and tracking

Programme attendance was measured by calculating the number of sessions attended (ranging from 1–16) over 6 months. Food tracking frequency was constructed from weekly food journals. It was assumed that participants who did not turn in the journals did not self-monitor their dietary intake.

#### Anthropometric measurements

Participant’s weight was measured using digital Weight Watchers scales. The scales were calibrated using a 20-pound weight on the scale. Waist circumference was measured with no more than one layer of light clothing using a tape measure wrapped around the waist in line with the umbilicus, to the nearest 0·1 inches. Participant’s height was measured without shoes for their standing height using a wall-mounted Seca digital stadiometer. BMI was calculated as follows: weight (kg)/height (meters squared). Participants were weighed at baseline, 6 months and at weekly sessions. The mean weight change was calculated from baseline to 6 months.

#### Clinical factors

HbA1c was measured at baseline and 6 months. Diabetes status (prediabetes *v*. T2D) was measured at baseline. BMI was calculated based on measured height and weight at baseline and 6 months.

#### Demographics

Data included age, gender, education, income and race/ethnicity.

#### Focus groups

All participants were invited to participate in focus groups, and twenty-two individuals accepted the invitation. Three focus groups were conducted by a trained qualitative researcher who consulted on the project. All participants were invited to attend the focus groups to provide qualitative feedback about the overall programme and experience with dietary tracking. A protocol was used to guide discussion, which prompted participants to share their feedback (see online supplementary material, Supplemental Table with questions is included). The focus groups were also part of the larger study and lasted 90 min.

### Data analyses

*Food Journal data:* Means were calculated on total nutrient intake at baseline (week 2) and 6 months (week 22) from weekly diet records. The ESHA’s Food Processor® Nutrition Analysis software was used to assess mean intake of nutrient composition as it provided a robust food and ingredient database (over 140 000) with an easy-to-use interface for accurate and comprehensive nutrition analysis. For reported foods that were not in the database (e.g. home-cooked meals), the ingredients and quantity provided in the journal were used to calculate the nutritional components. Macronutrients (carbohydrates, fibre, total fat, saturated fat, protein), total energies, Na and vitamin D were analysed. Average measurements were quantified using the appropriate units for the RDA. Macronutrients were analysed using the acceptable macronutrient distribution range. RDA was based on an 8368 kj (or 2000 kcal) diet, while the acceptable macronutrient distribution range percentages were calculated using the average kcals. Potential deficiencies in a nutrient were defined as less than or equal to 50 % of the RDA. Nutritional data from the ESHA software were transferred into a statistical software (Statistical Package for Social Sciences, SPSS 29) for data.

*For dietary behaviour,* descriptive statistics and univariate analysis were conducted first. We used intention-to-treat analysis and included all fifty-six participants who completed food journals, regardless of session attendance. Paired *t* test examined baseline to 6 months changes in dietary behaviour and mean nutrient intake among participants by gender. The multivariate regression model examined the association of dietary tracking and behaviour, nutrient intake, HbA1c and weight loss, controlling for demographic factors [gender, diabetes status and baseline BMI]. Education and income did not have a significant bivariate association with weight change and were excluded from the regression model due to the small sample size.

*Focus group data:* The focus group recordings were transcribed verbatim and coded in NVivo by two trained research assistants to ensure content accuracy. The coders used a hybrid inductive and deductive (or ‘theoretical’) coding approach to achieve the aims of this qualitative study^(29)^. Thematic analysis consisted of the researchers familiarising themselves with the transcripts followed by discussions to reconcile discrepancies in codes and collaboratively categorising codes to identify and define major themes.

## Results

### Descriptive statistics for the study population

A sample of ninety-four adults with T2D or prediabetes participated in the intervention. However, 56 (66 %) participants completed weekly food journals (optional) for 6 months (Table 2). The majority were females (73 %), had prediabetes (54 %), had an associate’s or college degree (51 %), and had an annual income of less than $50 000 (54 %). The mean age was 59·5 (sd 11·3) years, with a range of 35–83 years; the mean BMI was 36·2 (sd 7·3) kg/m^2^. Morbid obesity (BMI ≥ 35·0 kg/m^2^; not shown in table) was present in half of the males (53·3 %) and females (50 %). Mean baseline HbA1c was 8·0 % and 5·9 % for participants with T2D or prediabetes, respectively. Overall, mean attendance for programme sessions was 12·5 (sd 2·9) sessions (range 1–16) during the first 6 months of the programme. Participant characteristics are presented in Table 2.

**Table 2.** Demographic characteristics of the participants

Participants (*n* 56)	Mean	sd
Age (years)	59·5	11·3
BMI^†^	36·2	7·3
Waist circumference (inches)	42·6	6·4
Attendance of programme sessions	12·5	2·9
Baseline HbA1c		
T2D	8·0 %	1·4
Prediabetes	5·9 %	0·3
Baseline Weight (pounds)		
T2D	236·6	58·2
Prediabetes	207·1	43·7
	Percent	*n*
Female	73·2	41
Non-Hispanic White^**^	98	54
Education^**^		
High school/some college	13·5	7
Technical school	28·8	15
College graduate/professional degree	53·6	30
Status of chronic condition		
Diabetes	46·4	26
Prediabetes	53·6	30
Income (USD)^**^		
< 25 000	16·0	8
$25 000–$49 999	38·0	19
$50 000–$74 999	22·0	11
$75 000–$99 999	14·0	7
> $100 000	10·0	5

Sample size includes fifty-six adults with type 2 diabetes (T2D) or prediabetes.

† BMI = weight in kg/height in meters^2^.

** Numbers do not add up to the total due to missing values.

### Dietary self-efficacy, dietary intake, and nutrient composition

Table 3 shows the average dietary self-efficacy, intake of fruits and vegetables (servings/day), and % of fried food consumption in their diet. The highest possible self-efficacy score was 100, and the mean baseline score (75·7 (sd 14·8)) indicated relatively high dietary self-efficacy. The findings presented in Table 3 reveal that dietary self-efficacy improved after 6 months of the intervention (mean = 82·1 (sd 13·4); *P* = 0·02). The average servings of fruits and vegetables per day was relatively low at 2·3 (sd 1·5) since the recommendation is at least 5 servings per day. Intake of fruits and vegetables improved after the intervention (2·8 (sd 1·5); *P* = 0·02), and females reported greater dietary variety (*P* = 0·02). Participants also reported a reduction in fried food intake (%) at 6 months of the programme (*P* < 0·01).

**Table 3.** Baseline and 6-month programme changes in dietary self-efficacy, dietary behaviour and nutrient composition among participants by gender

	Total participants (*n* 56)					Programme change	Female (*n* 41)				Programme change	Male (*n* 15)				Programme change
	Baseline			6 months			Baseline		6 months			Baseline		6 months		
	Mean	sd		Mean	sd	*P*-value	Mean	sd	Mean	sd	*P*-value	Mean	sd	Mean	sd	*P*-value
Dietary Self-efficacy^†^	75·7	14·8		82·1	13·4	0·02	75·38	16·5	80·2	12·2	0·06	77·6	8·5	82·2	14·6	0·13
fruit and Vegetable Intake (servings)^‡^	2·3	1·5		2·8	1·5	0·02	2·06	0·7	2·8	1·09	0·02	1·8	0·5	2·00	0·8	0·06
Fried Food Intake %^§^	60	15·0		22	41·0	< 0·01	56·7	14·2	21·6	6·8	< 0·01	69·2	23·7	23·1	12·1	0·03
nutrient Intake																
Energies (kcal)	1440	491		1376	552	0·16	1393·7	476	1325·1	492	0·37	1566·5	526	1515·0	591	0·68
Protein (g)	64·4	23·2		64·7	27·4	0·92	64·6	23·6	63·97	23·2	0·88	64·1	22·8	66·8	25·9	0·56
Carbohydrates (g)	170·7	58		159·9	53	0·07	169·8	57	133·8	39	0·01	173·2	61	130·7	76	0·18
Fibre (g)*	16·9	6·0		17·7	6·0	0·11	17·3	5·8	17·5	5·6	0·42	16·0	7·0	18·4	7·1	0·02
Fat and cholesterol																
Fat (g)**	55·1	25·3		50·3	28·4	0·07	52·3	23·7	47·0	27·1	0·20	62·7	28·5	33·3	30·7	0·44
Cholesterol (mg)	188·0	94·8		181·1	91·6	0·40	199·9	100·5	188·1	94·3	0·36	143·3	102·6	124·1	83·2	0·26
Vitamins and minerals																
Na (mg)**	2645·6	856	1944·9		856	< 0·01	2783·0	852	1955·8	916	< 0·01	2868·8	856	2415·4	856	0·48
Vitamin D (µg)*	1·3	1·2	1·5		1·5	0·65	1·4	1·3	1·5	1·5	0·46	1·1	0·6	1·7	1·4	0·23

Baseline nutrient intake (mean values) was assessed from Week 2 food dairies and 6-month nutrient intake was assessed from Week 22 food dairies. Nutrient intakes and nutrient values reported are ‘per day’.

Sample size includes fifty-six adults with type 2 diabetes (T2D) or prediabetes.

* < 50 % RDA or less than the recommended range of intake of macronutrient (acceptable macronutrient distribution range; AMDR);

** > 100 % RDA or > AMDR.

† Dietary self-efficacy was assessed by a validated 20-item Eating Habits Confidence survey^(26)^ with a higher score indicating higher self-efficacy.

‡ Fruit and Vegetable intake was assessed by servings/day.

§ Self-reported deep-fried foods consumed per day, recategorised as >= 10 %.

The mean daily nutrient intake computed from food journals is shown in Table 3. Nutrient intakes and nutrient values reported are per day. Mean macronutrient intakes were carbohydrates 170·7 g, protein 64·4 g and fat 55·1 g, respectively, at the start of the programme. The average Na intake was over the recommended value of 2400 mg at baseline, i.e. 2645·6 (sd 856) mg. Similarly, the average dietary vitamin D level of 1·3 µg (sd 1·2) µg was below the daily recommendations. Mean baseline macronutrients for females and males were carbohydrates (169·8 g *v*. 173·2 g); protein (64·6 g *v*. 64·4 g) and fat (52·3 *v*. 62·7 g), respectively. Changes in micronutrient intake by participants were noted. Notably, participants had a lower intake of carbohydrates and fat (acceptable macronutrient distribution range) at 6 months of the programme. Females had a significant reduction in carbohydrate intake compared to their male counterparts (*P* = 0·01). Dietary Na intake also improved for all participants (*P* < 0·01), but significant reductions were noted for females (*P* =< 0·001); it reached the acceptable range of < 2400 mg. Dietary fibre intake did not meet the recommended levels at baseline or at 6 months (males > 30 gm *v*. 18·4 gm and females > 21 gm *v*. 17·5 gm).

### Dietary tracking, attendance and weight loss

Participants who consistently completed food tracking (i.e. 16 weeks of food journals) in the programme’s first 6 months were described as consistent trackers (58·9 %). Participants with dietary tracking attended an average of twelve sessions in 6 months (range 1–16); 66·1 % attended 11–16 sessions. Unadjusted subgroup comparisons showed no significant differences in programme attendance or weekly food tracking by gender. However, consistent trackers had significantly higher attendance in the weekly programme sessions. Completion of food journals encouraged participants to create measurable weekly goals as well as share their reflections at programme sessions. Participants with inconsistent diet tracking completed 9·5 weeks of food journals (*P* = 0·01) (Table 4). Average weight loss during the 6-month assessment period was 4·6 (sd 9·14) lbs., statistically similar among men (–4·45 (sd 7·7) lbs.) and women (–4·6 (sd 9·6) lbs.), but significantly higher among consistent dietary trackers (7·2 lbs) as compared with inconsistent trackers (< 1 lb., *P* < 0·01) (Table 4).

**Table 4.** Attendance, food tracking, dietary behaviour and changes in glycaemic level and weight

			Gender				*P*-value	Tracking status				*P*-value
	Total		Men		Women			Consistent		Inconsistent		
	Mean	sd	Mean	sd	Mean	sd		Mean	sd	Mean	sd	
*n*	56		15		41			33		23		
Number of sessions attended	12·5	2·9	12·0	2·4	12·6	3·0	0·84	15·8	8·9	9·5	8·1	0·01
Number of food records (weekly)*	13·1	9·0	11·1	9·9	13·8	8·7	0·34	15·8	8·9	9·5	8·1	0·01
FV servings/day^†^	2·9	1·2	2·3	1·1	2·8	1·4	0·04	3·0	1·4	2·92	1·1	0·74
6-Month weight change, lbs**	−4·6	9·1	−4·5	7·7	−4·6	9·6	0·95	−7·2	9·8	−0·8	6·4	< 0·01
6-Month HbA1c % change**	−3·1	5·1	−3·7	5·6	−2·8	4·9	0·62	−3·1	5·2	−3·0	4·9	0·97

*P*-value = difference between groups; *Number of complete weekly food journals provided for review by their Health Coaches during the study period (6 months). ^†^FV = Fruit & Vegetable intake was assessed by servings/day. **Weight loss (lbs) was assessed by the difference in 6-month and baseline value and HbA1c % change was assessed by the difference in 6-month and baseline value divided by baseline value. Sample size includes fifty-six adults with type 2 diabetes (T2D) or prediabetes.

### The association between weight loss, nutrient intake and demographic characteristics

Factors associated with participants’ weight loss at 6 months are shown in Table 4. Predictors in the multiple linear regression model included gender, disease status (diabetes *v*. prediabetes), baseline BMI and HbA1c, dietary self-efficacy, number of sessions attended, dietary tracking (consistent *v*. inconsistent) status and macronutrient intake and energies at the start of the programme. Significant predictors in the regression model included DPM session attendance, consistent dietary tracking, baseline HbA1c, baseline nutrients and disease status. At 6 months, participants who consistently tracked their food and completed food journals had higher weight loss. Similarly, a higher intake of energies but a lower intake of carbohydrates at the start of the programme resulted in higher weight loss (*P* < 0·01; Table 5), while higher baseline HbA1c was also associated with greater weight loss. Participants with an annual household income of < $50 000 had greater weight loss (*P* = 0·01), whereas participating in fewer DPM sessions was linked to less weight loss (*P* = 0·03). The overall model was significant (F = 3·22, *P* < 0·01), accounting for 43·5 % of the variance in weight loss.

**Table 5.** Factors associated with weight loss in the DPM study (*n* 56)

Predictors	Standardised coefficient (Beta) estimate^†^	95 % CI	*P*-value
Attendance	0·27	0·06, 1·61	0·03
Energies (kcal)	−1·36	−0·03, –0·008	< 0·01
Carbohydrates (g)	1·19	0·07, 0·26	< 0·01
Protein (g)	0·35	−0·04, 0·29	0·14
HbA1c	0·42	0·29, 4·80	0·02
Tracking status (consistent)	−0·41	−11·3, –2·4	< 0·01
Diabetes status	0·43	1·02, 13·17	0·02
Gender (male)	0·03	−5·09, 4·08	0·82
Education*	−0·24	−3·58, 0·21	0·08
Income**	0·38	0·66, 4·62	0·01
Dietary self-efficacy***	−0·19	−0·41, 0·10	0·23

Multivariate regression model examined intervention effects on weight loss. Socio-demographic factors included gender, status (diabetes *v*. prediabetes) and baseline HbA1c. Attendance included the number of Diabetes Prevention and Management program (DPM) sessions attended in 6 months. Food journal tracking included consistent *v*. inconsistent trackers. Macronutrient intake included baseline fat, protein, carbohydrates and total energies.

Sample size includes fifty-six adults with type 2 diabetes (T2D) or prediabetes.

* Education - reference category was college education.

** Income – reference category was an annual income of $50 000 or higher.

*** Dietary self-efficacy was assessed by a validated twenty-item Eating Habits Confidence survey^(26)^ with a higher score indicating higher self-efficacy.

† A negative parameter estimate indicates that an increase in the measure predicts greater weight loss.

### Focus group results

Twenty-two participants from the two sites participated in three focus groups after the conclusion of the programme (female 77 %, employed full-time 64 %, married 59 %, annual income < $50 000 and T2D status 59 %). As noted, this study was part of a larger study, and only focus group themes about programme feedback and experience with dietary tracking are reported here.

Several themes emerged from the data. First, the programme was comprehensive and gave useful tools, not just information. ‘The thing I noticed about this programme compared to others is that it wasn’t just about eating … I think they did an excellent job adding exercise and other things.’ Group support was believed to be the most important element for success. A female with diabetes commented, ‘I thought this would be a good opportunity to join a group where I could lose weight and have healthy eating habits and everything.’ Second, the programme improved eating habits. A male with prediabetes noted, ‘I cut out pasta in the last two weeks and lost weight. I’m not diabetic, but when I quit drinking, I started gaining weight. All I did so far was cut out pasta.’ Third, most participants agreed that it was difficult to keep food journals. ‘I think it was easier at first; everything was new, shiny, and bright, and I filled out everything. Now, it’s difficult to do it every day.’ ‘I missed a few days, and it was hard to catch up. I started to resent those books [logs].’ Fourth, although food tracking was cumbersome, it was a valuable tool. ‘The logs that we keep allow us to look back and see what’s worked and what hasn’t, especially if we’re weighing ourselves daily.’ ‘The logs that we keep allow us to look back and see what’s worked and what hasn’t worked, especially if we’re weighing ourselves daily.’ Another stated, ‘It pays to be honest with recording your foods, fats, and energies. Although I don’t like it a bit [food tracking], it helps. Overall, it’s a good thing.’ Logging the food daily also helped one woman to make better choices. ‘I am more conscious of the programme and food and thinking, ‘Is the doughnut really worth it?’ The programme has made me more conscious of everything I put in my mouth. Fifth, consistency with tracking is key. ‘I hate them [log books], but I did them anyway. Doing it regularly is what got me to where I am with losing weight and stuff. I’m being honest.’ ‘If I missed a few days, I had to play catch up and became frustrated.’

## Discussion

This community-based, multicomponent lifestyle intervention was associated with improved dietary tracking and dietary behaviours in adults living in rural areas. While food journals and dietary tracking are useful strategies to improve dietary habits, long-term (6-month) dietary tracking with tailored feedback by HC is novel and not currently implemented in DSME programmes. Additionally, tracking adherence serves as a key indicator of how effectively obese adults with T2D or prediabetes are motivated and engaged in making healthy dietary changes. It also enables the assessment of changes in micronutrient intake and dietary behaviours. Findings from the qualitative focus groups aligned with the self-reported surveys, anthropometric data (weight) and clinical outcomes (HbA1c), showing increased consumption of fruits and vegetables and a reduction in fried foods in participants’ daily diets. In other words, participants reported an increase in dietary variety. Participants with consistent weekly dietary tracking had significantly higher programme engagement and weight loss than inconsistent trackers. This finding indicates that emphasising and encouraging dietary tracking can improve the effectiveness of nutrition education and lifestyle interventions in rural and limited resource settings. Significant improvement in dietary self-efficacy was only noted among individuals with prediabetes, who reported they were the most confident in their ability to change their dietary habits by participating in the programme.

While health coaching and peer support strategies are used to help people maintain healthy behaviours in diabetes and weight loss programmes^(28)^, this is the first community trial to examine the impact of an HC-led DPM intervention in rural settings. Qualitative focus groups showed the programme was deemed acceptable and benefited both adults with T2D or prediabetes. Participants reported a few programme components that were most helpful in improving nutrition behaviour and disease self-management. Participants had poor dietary habits before they started the intervention^(30)^, but food journals and dietary tracking offered a successful strategy to improve their dietary habits^(31,32)^. Although we did not directly examine eating patterns of participants, however, consistently monitoring what they eat through dietary tracking could be a helpful strategy for maintaining healthy eating habits even during times when there are temptations to overindulge, such as the holiday season. This resulted in sustained and significant weight loss as compared to participants with inconsistent/rare dietary tracking. Hence, future behavioural interventions should emphasise the benefits of dietary self-monitoring and tracking in rural Appalachian states.

The programme encouraged behaviour changes at various levels of the dysglycemic spectrum that improved the overall dietary intake. For example, findings showed reductions in macronutrients such as fat intake, dietary cholesterol and Na intake that benefited participants with both prediabetes or T2D. These reductions were noteworthy as they helped in lowering blood pressure and risks for CVD and chronic kidney disease^(33)^. Further, dietary advice delivered by trained HC was vital for reinforcing healthy dietary habits for programme effectiveness and could be used in programmes in resource-poor settings where interventions are unavailable to area residents. DSMES programmes have been found to be efficacious for health behavioural changes and diabetes outcomes^(5,34,35)^. Therefore, access to these programmes can benefit WV adults with suboptimal social determinants of health factors (e.g. lack of transportation, food access and food deserts). Alleviating access to a setting they trust (churches) during the weekends and using the traditional dietary tracking method seemed to optimise dietary tracking and attendance. In addition, participants liked the interactive dietary sessions that provided skill-building tasks around food measurement, traditional Appalachian dietary habits and cultural norms and low-cost, locally available seasonal food items with macronutrient quality and avoidance of fat and processed foods^(12,36,37)^ Weekly tracking and feedback by HC reinforced accountability, encouraged healthier dietary modification and concurred with nutrition and lifestyle changes^(38)^. This educational model can be expanded and integrated into clinics as 70 % of WV is considered health professional shortage areas for diabetes and nutrition education.

Consistent with our expectations and evidence, community-based lifestyle interventions have been successful in rural areas^(39,40)^, but attrition rates are generally high (∼50 %)^(41)^. However, the successful connection of participants with HCs and programme personnel and weekly follow-up sessions improved engagement and retention. The current study found that programme attendance improved food tracking due to improved knowledge and reinforcement of culturally adapted dietary strategies^(42)^. However, participants also learned from peers who became members of their social network. Knowledge was generally low about nutrient composition and content of food that improved with dietary tracking and feedback from HC for modifications to lower energies, Na and fat content in their diet. A recent meta-analysis of DSME programmes showed that DSME interventions integrated with peer support effectively enhances glycaemic control in T2D patients^(43)^.

This study builds on the research team’s success with culturally tailored DPM programmes designed for rural adults^(17,18)^. The strength includes longitudinal data to compare changes in behavioural, anthropometric and clinical factors over 6 months. Use of 7-day food diaries and partnership with churches for programme implementation. The use of low-cost, trained HC who were part of the local Appalachian culture helped engage hard-to-reach individuals with limited health literacy and financial/medical resources. Adherence to a healthy diet is essential for long-term metabolic control and improved quality of life^(44)^ which benefits healthy eating in rural Appalachians^(45,46)^. Rural residents exhibit healthcare-avoidant behaviours related to the Appalachian culture of distrust^(47)^. In addition, patient-level factors (e.g. lower literacy, education, income), psychosocial factors (e.g. poor disease coping, mental well-being, and social support)^(47,48)^and limited access to DSMES/infrequent and ineffective provider counselling have been noted^(49–51)^ Hence, innovations of integrating education into patient portals and the use of self-management apps to track dietary behaviour could reduce some patient-level barriers as well as time constraints of providers and should be investigated in future programmes^(52)^. However, challenges for weekly tracking should be taken into account, which included time commitment, forgetting to log foods, recording nutrient composition using the CalorieKing book, nutrition facts panel of packaged foods or other sources (internet, etc.) among rural residents with limited digital and health literacy^(53)^.

The generalisability of the results should be approached carefully due to several limitations of this study. The results are based on 6-month dietary tracking with a small sample size. In addition, there was no usual care or control group, with the majority of participants being non-Hispanic Whites (97 % of the WV population), limiting the generalisability of our findings to diverse rural adults with T2D or prediabetes. Also, dietary tracking is based on self-reporting. Thus, recall bias might affect the accuracy of their intake. In addition, including participants who completed weekly food diaries has the potential for selection bias of motivated participants. HCs’ weekly interactions, counselling style and engagement could have affected healthy dietary modifications and should be explored in future studies. Although the study was conducted in 2015–2017, our findings are relevant for understanding rural adults dietary tracking and behaviour.

### Conclusions

Rural adults with T2D or prediabetes who consistently tracked their diet had greater weight loss and improved dietary self-efficacy and intake over 6 months. The DPM programme was effective in engaging two-thirds of participants to complete food journals for 6 months of the programme. Although the findings showed a modest decrease in weight, the study has several notable strengths that make a unique contribution to the literature about the effectiveness of a trained HC-delivered multicomponent intervention in rural populations. Qualitative feedback reported by participants included improvement in healthier eating habits; consistency of dietary tracking was cumbersome yet was beneficial for making better choices and being honest. Future studies should explore programme effectiveness in larger and diverse racial/ethnic rural participants. Further, an HC-led lifestyle programme may be a promising approach to reducing diabetes disparities in rural areas.

## Supporting information

Misra and James supplementary material Misra and James supplementary material

## Supplementary material

For supplementary material accompanying this paper visit https://doi.org/10.1017/S1368980025000436
