# Supplementary material for: The role of dietary tracking on changes in dietary behaviour in a community-based diabetes prevention and management intervention
Source: Public Health Nutr. 2025 Mar 27;28(1):e73. doi: 10.1017/S1368980025000436 (PMC12086725; doi:10.1017/S1368980025000436)
Supplement: Misra and James supplementary material [file S1368980025000436sup001.docx]

# **Supplemental Table**

# DPM Program - Focus Group Guide

**Time of Interview:** 60-90 Minutes

# Focus Group Questions

# Core Questions

1. What comes to mind when you think of diabetes?
   1. Probe 1. How has your view of diabetes changed since you participated in the program?
2. Why did you decide to be involved with the diabetes program?
   1. Probe 1. What did you want to get out of the program?
      1. Were those needs met?
         1. If not, how could they have been met?
3. What challenges did you experience with the program?
   1. Probe 1. What barriers (if any) made it difficult for you to attend the sessions?
      1. What about transportation, time the sessions were scheduled, weather, etc.?
   2. Probe 2. What could the program have done to minimize those challenges?
4. What changes (if any) would you like to see in the program?
   1. Probe 1. What about the day, time, and location?
   2. Probe 2. Were the meeting times too frequent or not frequent enough?
   3. Probe 3. Were the incentives or payment adequate?
5. Do you have any other comments or suggestions on how we can modify or improve the program?

# Group Specific Questions

1. Please describe your overall experience in participating in the program.
   1. Probe 1. How has the program helped you?
      1. How has your diet changed?
      2. How has your activity level changed?
      3. How has your weight changed?
   2. Probe 2. Is there anything that has improved in your life other than your eating and activity level?
2. What kept you motivated to keep attending the sessions?
   1. Probe 1. What about a sense of obligation, family support, or new friendships within the program?
3. What are three useful things that you have learned from the program?
   1. Probe 1. How are you using or practicing them?
4. Please describe your experience of the weekly contact with your health coach?
   1. Probe 1. Were there any things that were annoying? If so, what?
   2. Probe 2. What are some of the biggest ways that the coach helped?
5. Please describe your experience in completing the tracking booklets each week?
   1. Probe 1. What was the biggest challenge?
